# Predictors of in-hospital mortality in elderly patients with heart failure treated with tolvaptan

**DOI:** 10.20407/fmj.2021-027

**Published:** 2022-07-22

**Authors:** Masakazu Kobayashi, Mutsuharu Hayashi, Ryo Yamada, Tomoya Ishiguro, Wakaya Fujiwara, Hideki Ishii, Hiroyuki Naruse, Eiichi Watanabe, Yukio Ozaki, Hideo Izawa

**Affiliations:** 1 Department of Medicine, Division of Cardiology, Fujita Health University Okazaki Medical Center, Okazaki, Aichi, Japan; 2 Department of Cardiology, Fujita Health University, School of Medicine, Toyoake, Aichi, Japan; 3 Department of Cardiology, Fujita Health University Bantane Hospital, Nagoya, Aichi, Japan

**Keywords:** Heart failure, Elderly, Tolvaptan, Early initiation

## Abstract

**Objectives:**

We conducted an analysis of first-time tolvaptan users (≥80 years old) to determine the factors associated with the prognosis of elderly patients with heart failure.

**Methods:**

We retrospectively analyzed 66 consecutive patients with worsening heart failure (aged ≥80 years) who were admitted to Fujita Health University Bantane Hospital from 2011 to 2016 and treated with tolvaptan. Differences between the in-hospital death and survival groups were evaluated. Multivariate logistic regression analysis was also performed to identify the risk factors for mortality.

**Results:**

Sixty-six patients were included, and 26 patients died during the index hospitalization. The patients who died had a significantly higher prevalence of ischemic heart disease; a higher heart rate; higher levels of plasma C-reactive protein, blood urea nitrogen (BUN), and creatinine; a lower serum albumin level; and a lower estimated glomerular filtration rate than surviving patients. The proportion of patients requiring early initiation of tolvaptan treatment (within 3 days of admission) was significantly higher in surviving patients. On the basis of multivariate logistic regression analysis, although a high heart rate and high BUN levels were independent factors for in-hospital prognosis, they were not significantly associated with the early use of tolvaptan (≤3 days vs. ≥4 days; odds ratio=0.39; 95% confidence interval=0.07–2.21; p=0.29).

**Conclusions:**

This study revealed that a higher heart rate and higher BUN levels were independent factors for in-hospital prognosis in elderly patients who received tolvaptan and that early tolvaptan use may not always be effective in elderly patients.

## Introduction

Japan is one of the most rapidly aging countries in the world. As the population has aged, the number of patients with heart failure has increased, and this trend is expected to accelerate.^1,2^ Heart failure in the elderly is characterized by a high prevalence of comorbid systemic factors, such as infection, anemia, renal failure, stroke, and malignant diseases.^3–5^ These comorbidities, rather than cardiac function itself, are the main determinants of the prognosis in elderly patients with heart failure. The prognosis of elderly patients with heart failure is poor, and selection of the optimal treatment for these patients is important.

Tolvaptan, a vasopressin V2 receptor antagonist, exerts a purely diuretic effect by blocking vasopressin V2 receptors in the medullary collecting duct.^6^ A randomized trial of tolvaptan versus placebo in patients with acute decompensated heart failure found that the drug improved congestion but not long-term prognosis.^7^ Conversely, a Japanese study of elderly patients with heart failure reported that tolvaptan improved patient prognosis when treatment was started within 3 days of hospital admission.^8^ However, information on the prognostic value of tolvaptan in elderly patients with heart failure is insufficient. Therefore, we conducted an analysis of first-time tolvaptan users among patients aged 80 years or older who were admitted to our affiliated hospital with congestive heart failure to identify the factors associated with prognosis.

## Materials and Methods

We retrospectively analyzed 66 consecutive patients with worsening heart failure aged 80 years or older who were admitted to Fujita Health University Bantane Hospital from 2011 to 2016 and treated with tolvaptan for the first time. Tolvaptan is usually given to patients with signs of worsening heart failure such as jugular venous distention, lower-limb edema, or pulmonary congestion even though they had already been given ≥40 mg of furosemide. Worsening heart failure was defined as new-onset heart failure or worsening chronic heart failure. The diagnosis of heart failure was based on the Framingham criteria for the clinical diagnosis of heart failure.^9^ The signs of volume overload (rales, jugular venous distention, and/or ankle edema) were identified in all patients by the attending physician, and laboratory measurements and echocardiography were performed in all patients prior to the initiation of tolvaptan treatment. The diagnosis of dilated cardiomyopathy was based on the definition of the World Health Organization/International Society and Federation of Cardiology Task Force.^10^ Because of their advanced age, detailed information on the coronary artery condition of many patients was not available, and many had a combination of factors, such as hypertensive heart disease, valvular disease, and atrial fibrillation, as the causes of heart failure. These patients were then classified as “others.”

This study was conducted in accordance with the Declaration of Helsinki. Written informed consent was obtained from all participants, and the ethics committee of our institution approved the study and chart review protocol (ID: HM20-161).

All statistical analyses were performed using JMP Pro 15 (SAS Institute Inc., Cary, NC, USA). Continuous variables were presented as the mean and standard deviation or the median and interquartile interval. Normality was tested using the Shapiro–Wilk test. On the basis of their distributions, the differences between the groups were evaluated using Student’s unpaired *t*-test or the Wilcoxon’s rank-sum test. Categorical variables were presented as numbers and percentages and compared using the chi-squared test or Fisher’s exact test. Univariate logistic regression analysis was performed to identify the candidate predictors of in-hospital mortality. The variables with a value of p<0.05 in univariate analysis were then included in a multivariate logistic regression model to identify the independent predictors of in-hospital death. Odds ratios (ORs) and 95% confidence intervals (CIs) were calculated. A value of p<0.05 was considered statistically significant.

## Results

Sixty-six patients (38 men, 58%) were consecutively enrolled in our study. The median patient age was 88 years (interquartile range, 83–91). Among all patients, the prevalence rates of hypertension, diabetes, and dyslipidemia were 76%, 26%, and 30%, respectively. Of all patients, 22 (33%) had a previous history of myocardial infarction.

Forty patients (61%) were alive at discharge, but 26 patients (39%) died during the index hospitalization. Table 1 presents the baseline demographic and clinical characteristics of the two groups. No significant differences in age, sex, or medications (e.g., diuretics) between were observed between the in-hospital survival and death groups. Myocardial infarction was significantly more common in patients who died during the index hospitalization. The heart rate was significantly higher in patients who died than in those who survived. On the basis of the blood test results, the patients who died had statistically significantly higher plasma C-reactive protein (CRP), blood urea nitrogen (BUN), and creatinine levels; a lower serum albumin level; and a lower estimated glomerular filtration rate than those who survived.

After hospital admission, the median time to start tolvaptan treatment was 6.5 days (interquartile range, 1.75–17). Twenty-three patients (35%) started tolvaptan treatment by day 3 (early initiation). The proportion of patients with early initiation of tolvaptan treatment was significantly higher in surviving patients than in patients who died during the index hospitalization. The early initiation of tolvaptan treatment was associated with a relatively younger age (median, 85 years [interquartile range, 81–89] vs. 88 years [interquartile range, 84–93]; p=0.02), higher systolic blood pressure (mean, 127 mmHg [SD, 24] vs. 112 mmHg [SD, 20]; p=0.012), lower plasma CRP levels (median, 0.50 mg/dL [interquartile range, 0.18–3.86] vs. 4.58 mg/dL [interquartile range, 1.23–9.55]; p<0.001), and higher serum albumin levels (median, 3.2 g/dL [interquartile range, 3.0–3.6] vs. 2.8 g/dL [interquartile range, 2.4–3.1]; p<0.001).

Table 2 presents the results of univariate and multivariate logistic regression analyses for in-hospital survival. In the multivariate model, heart rate (per 10 beats/min, OR=1.93; 95% CI=1.25–3.34; p=0.007) and BUN levels (OR=1.05; 95% CI=1.01–1.10; p=0.02) were determinants of in-hospital death. The early initiation of tolvaptan treatment was not significantly associated with in-hospital death (≤3 days vs. ≥4 days, OR=0.39; 95% CI=0.07–2.21; p=0.29).

## Discussion

In the present study, we conducted a retrospective analysis of elderly patients with heart failure who received tolvaptan during hospitalization at our affiliated hospital to identify the factors associated with prognosis.

In our cohort, patients who died during hospitalization had a higher heart failure rate because of ischemic heart disease, a higher heart rate, a lower left ventricular ejection fraction, higher CRP levels, lower albumin levels, and worse renal function than those who were discharged alive. The early initiation of tolvaptan treatment (within 3 days of admission) was also associated with lower mortality, but this association with death was not significant in multivariate analysis. A higher heart rate and higher BUN levels were the independent factors for in-hospital prognosis in elderly patients treated with tolvaptan.

In the acute phase of heart failure, achieving early decongestion and dyspnea relief is important. In the elderly, prolonged bed rest is likely to lead to disuse and poor outcomes; therefore, early decongestion of congestive heart failure is more important in elderly patients than in younger patients. Moreover, many elderly patients have impaired renal function. The early administration of tolvaptan after hospital admission in elderly patients with heart failure has proven beneficial with fewer adverse effects on renal function.^11–13^ Although a double-blind randomized clinical trial of tolvaptan revealed no benefits regarding long-term prognosis, the effects of tolvaptan on short-term outcomes such as death during hospitalization, as examined in this study, have not been fully elucidated. Matsukawa et al.^8^ reported that the early use of tolvaptan (up to 3 days after admission) shortened hospital stay and reduced hospital mortality in elderly patients with heart failure aged 75 years or older. In our study of older patients, fewer deaths were observed in patients with the early tolvaptan use. In multivariate analysis, a higher heart rate and higher BUN levels were associated with death during hospitalization. A higher heart rate and higher BUN could indicate hemodynamic insufficiency, and the observed associations of a higher heart rate and higher BUN levels with prognosis are consistent with previous reports.^14–16^ Anemia has been reported to be associated with long-term prognosis in patients with heart failure.^17^ In the current study, anemia was not associated with hospital mortality. Further prospective studies with larger numbers of patients are needed to determine whether anemia is a prognostic factor for short-term outcomes, such as death during hospitalization, in this study. Early tolvaptan use was not associated with in-hospital death in this study. This finding suggested that tolvaptan treatment may not be effective in patients with concomitant infection, malnutrition, or dehydration, and treatment strategies other than early tolvaptan use should be considered. In fact, patients who started tolvaptan late were significantly older and they had higher plasma CRP levels and lower serum albumin concentrations than those who started treatment early. In elderly patients with heart failure with unstable oral intake or infection, tolvaptan use can cause adverse events, such as dehydration and hypernatremia, and patients who had a lower heart rate, lower BUN levels, lower plasma CRP levels, and higher serum albumin levels, all of which could indicate a good general condition, and early tolvaptan use resulted in a higher number of patients being discharged alive, even among those older than 80 years. This might mean that patients with fewer noncardiac comorbidities might benefit from the early use of tolvaptan, even if they are older than 80 years. Furthermore, although the early use of tolvaptan may be effective, it should be carefully considered in elderly patients older than 80 years, who often have a poor condition. Additionally, to clarify the efficacy of early tolvaptan use in elderly patients with multiple complications, larger prospective studies are needed.

### Limitations

This was a single-center study with a small number of patients. Furthermore, many of the deaths were caused by complex factors other than heart failure or were closer to “of natural causes.” This makes it difficult to conduct a detailed analysis of the cause of death. Therefore, the optimal dose and timing of tolvaptan administration should be evaluated in larger prospective studies. In addition, multiple regression analysis was performed to identify predictors of in-hospital mortality using a relatively large number of independent variables. In addition, the results of multiple regression analysis in this study need to be interpreted carefully.

In conclusion, this study revealed that early tolvaptan use may not always be effective in patients older than 80 years and that a higher heart rate and higher BUN levels were the independent factors for the in-hospital prognosis. Furthermore, we should consider treatment strategies other than the early use of tolvaptan in elderly patients with a poor condition.

## Figures and Tables

**Table1:** Patient characteristics

	Survived (n=40)	Died (n=26)	p value
Age (years)	87 (82–91)	88 (84–91)	0.25
Male (%)	24 (60)	14 (54)	0.62
Body weight (kg)	48.1 (41.4–58.2)	49.3 (43.4–57.0)	0.86
Body mass index (kg/m^2^)	22.0 (20.2–25.2)	21.1 (18.9–23.4)	0.16
LVEF (%)	56 (15)	46 (20)	0.023
Etiology			0.003
DCM (%)	6 (15)	3 (12)	
IHD (%)	7 (18)	15 (58)	
Others (%)	27 (68)	8 (31)	
Hypertension (%)	29 (73)	21 (81)	0.44
Diabetes mellitus (%)	8 (20)	9 (35)	0.18
Dyslipidemia (%)	11 (28)	9 (35)	0.54
Systolic BP (mmHg)	121 (23)	112 (22)	0.14
Diastolic BP (mmHg)	64 (18)	64 (15)	0.95
Heart rate (bpm)	74 (17)	89 (17)	0.0012
Medications			
Loop diuretics (%)	34 (85)	18 (69)	0.14
Beta-blocker (%)	23 (58)	10 (38)	0.13
ACE-I/ARB (%)	22 (55)	14 (54)	0.93
MRA (%)	4 (10)	4 (15)	0.70
Laboratory data			
Hemoglobin (g/dL)	10.7 (1.8)	10.3 (1.3)	0.36
CRP (mg/dL)	1.61 (0.24–4.22)	4.33 (0.87–9.04)	0.016
BUN (mg/dL)	22.0 (16.3–35.8)	41.0 (26.0–66.0)	0.003
Creatinine (mg/dL)	1.12 (0.83–1.42)	1.69 (0.95–2.00)	0.033
eGFR (mL/min/1.73 m^2^)	39.6 (30.2–60.0)	32.6 (20.9–56.8)	0.038
Na (mEq/L)	140 (132–144)	137 (131–142)	0.31
K (mEq/L)	4.2 (0.6)	4.2 (0.7)	0.42
Cl (mEq/L)	101 (6)	100 (7)	0.72
Albumin (g/dL)	3.1 (2.8–3.4)	2.7 (2.0–3.1)	0.004
Total cholesterol (mg/dL)	171 (41)	149 (29)	0.10
NT-pro BNP (pg/mL)	5940 (2738–16339)	10386 (3369–18430)	0.20
Early initiation of tolvaptan treatment (≤3 days)	19 (48)	4 (15)	0.008

ACE-I, angiotensin-converting enzyme inhibitor; ARB, angiotensin II receptor blocker; BP, blood pressure; BUN, blood urea nitrogen; CRP, C-reactive protein; DCM, dilated cardiomyopathy; eGFR, estimated glomerular filtration rate; IHD, ischemic heart disease; LVEF, left ventricular ejection fraction; MRA, mineralocorticoid receptor antagonist; NT-pro BNP, N-terminal pro-brain natriuretic peptide

**Table2:** Multivariate logistic regression model to determine the factors associated with in-hospital death

Independent variables	Univariate analysis			Multivariate analysis		
	Odds ratio	95% confidence interval	p value	Odds ratio	95% confidence interval	p value
Age (per 10 years)	1.49	0.57–4.00	0.42			
Male (vs. female)	0.78	0.28–2.12	0.62			
Etiology (IHD vs. others)	7.23	2.28–25.42	<0.001	6.74	0.95–47.76	0.11
LVEF (%)	0.97	0.93–1.00	0.03	0.99	0.93–1.04	0.65
Heart rate (per 10 bpm)	1.63	1.21–2.32	0.003	1.93	1.25–3.34	0.007
CRP (mg/dL)	1.12	1.02–1.26	0.03	1.03	0.92–1.16	0.61
BUN (mg/dL)	1.04	1.02–1.08	0.002	1.05	1.01–1.10	0.02
Creatinine (mg/dL)	1.66	0.97–3.13	0.08			
eGFR (mL/min/1.73 m^2^)	0.98	0.96–1.00	0.13			
Albumin (g/dL)	0.22	0.07–0.56	0.004	0.61	0.11–3.23	0.56
Total cholesterol (g/dL)	0.98	0.96–1.00	0.10			
NT-pro BNP (per 100 pg/mL)	1.00	1.00–1.01	0.08			
Early initiation of tolvaptan treatment (≤3 days vs. ≥4 days)	0.20	0.06–0.69	0.01	0.39	0.07–2.21	0.29

bpm, beats per minute; BUN, blood urea nitrogen; CRP, C-reactive protein; eGFR, estimated glomerular filtration rate; IHD, ischemic heart disease; LVEF, left ventricular ejection fraction; NT-pro BNP, N-terminal pro-brain natriuretic peptide
